# C-Reactive Protein for Predicting Prognosis and Its Gender-Specific Associations with Diabetes Mellitus and Hypertension in the Development of Coronary Artery Spasm

**DOI:** 10.1371/journal.pone.0077655

**Published:** 2013-10-28

**Authors:** Ming-Jui Hung, Kuang-Hung Hsu, Wei-Syun Hu, Nen-Chung Chang, Ming-Yow Hung

**Affiliations:** 1 Department of Cardiology, Chang Gung Memorial Hospital, Keelung, Chang Gung University College of Medicine, Taoyuan, Taiwan; 2 Laboratory for Epidemiology, Department of Health Care Management and Healthy Aging Research Center, Chang Gung University, Taoyuan, Taiwan; 3 Division of Cardiology, Department of Internal Medicine, Shuang Ho Hospital, Taipei Medical University, New Taipei City, Taiwan; 4 Division of Cardiology, Department of Internal Medicine, Taipei Medical University Hospital, Taipei, Taiwan; 5 Graduate Institute of Clinical Medical Sciences, Chang Gung University College of Medicine, Taoyuan, Taiwan; 6 Department of Internal Medicine, School of Medicine, College of Medicine, Taipei Medical University, Taipei, Taiwan; Yokohama City University School of Medicine, Japan

## Abstract

**Background:**

While hypertension is negatively associated with coronary artery spasm (CAS), scarce data are available on diabetes mellitus in relation to CAS. In addition, outcome prediction in patients with CAS is challenging due to the lack of appropriate biomarkers. Therefore, we sought to identify the roles that gender, high-sensitivity C-reactive protein (hs-CRP), diabetes mellitus and hypertension play in CAS development and prognosis.

**Methodology/Prinicpal Findings:**

Patients (350 women and 547 men) undergoing diagnostic coronary angiography with or without proven CAS but without obstructive stenosis were evaluated at long-term follow-up (median 102 months). Diabetic women and diabetic men with low hs-CRP levels had a low and high risk of CAS (odds ratio [OR]: 0.16, 95% confidence interval [CI]: 0.01–1.88 and OR: 5.02, 95% CI: 1.03–24.54, respectively). The ORs of CAS in both women and men with the highest hs-CRP tertile (>3 mg/L) reduced from 4.41 to 1.45 and 2.98 to 1.52, respectively, if they had diabetes mellitus, and from 9.68 to 2.43 and 2.60 to 1.75, respectively, if they had hypertension. Hypertension had a more negative effect on CAS development in diabetic than non-diabetic women, which was not observed in men. The highest hs-CRP tertile was an independent predictor of adverse outcomes. Patients with the highest hs-CRP tertile had more coronary events than patients with the lowest hs-CRP tertitle (p* = *0.021, log-rank test).

**Conclusions:**

Diabetes mellitus contributes to CAS development in men with low hs-CRP levels, but not in women. There are negative effects of diabetes mellitus and hypertension on CAS development in patients with high hs-CRP levels and especially in women. Elevated hs-CRP level independently predicts adverse outcomes.

## Introduction

Coronary artery spasm (CAS), associated with transient increase in troponin release [1], is an important cause of variant angina and ischemic heart disease [2]. Although diabetes mellitus has been shown to be a risk factor for developing cerebral spasm [3], it is not clear whether the disease plays a role in the development of CAS. While vasoconstriction is impaired in diabetes mellitus [4], vasodilatation has been shown to be impaired in type 2 diabetes mellitus and hypertension [5]. Diabetes mellitus and hypertension are recognized as modifiable risk factors for coronary artery disease (CAD) [6], both of the diseases, however, are found more frequently in patients with classic angina than in patients with vasospastic angina [7], [8]. While coronary vascular resistance increases in hypertension [9], [10], acute hypertension and increased coronary flow attenuate coronary vasoconstriction [11]. Furthermore, acetylcholine has been demonstrated to elicit a greater effect on aortic relaxation in female than in male hypertensive rats, indicating that there is a gender-dependent effect on endothelium-dependent nitric oxide-mediated vasorelaxation [12]. Collectively, these observations suggest that the effects of diabetes mellitus and hypertension on CAS may differ from their effects on CAD and between genders.

CAS is an inflammatory disease characterized by the presence of elevated high-sensitivity C-reactive protein (hs-CRP) [2], [13]. While the prognosis of patients with CAS is considered to be good [13], recurrent episodes of angina are frequently observed [14]. Although the associations between C-reactive protein and CAD is similar across diabetes status [15] and C-reactive protein is an independent prognostic marker of CAD [16], [17], the roles C-reactive protein play in CAS development in diabetic patients and in predicting prognosis of CAS have not been evaluated. Moreover, it has been demonstrated that glycated hemoglobin is not responsible for the impaired coronary vasodilatation associated with diabetes mellitus [18]. Previous studies have demonstrated that age contributes to CAS development in men, the risk impact of hs-CRP in CAS development differ between women and men and hypertension is negatively associated with CAS [2], [7], [13], [19]. We, therefore, aimed to determine the extent to which gender, hs-CRP, diabetes mellitus and hypertension affect the development and to what extent any association of hs-CRP on prognosis was independent of possible prognostic factors of CAS.

## Materials and Methods

### Ethics Statement

This study was approved by the Chang Gung Memorial Hospital Institutional Review Board (96–1069B) and Taipei Medical University-Joint Institutional Review Board (No. 201011004 version 1.2). All patients gave written informed consent.

### Study Population

From January 1999 to June 2011, 897 patients with suspected ischemic heart disease and no angiographic evidence of obstructive CAD were subjected to intracoronary methylergonovine testing. Inclusion criteria for patients with CAS included spontaneous chest pain at rest associated with ST-segment elevation or depression on electrocardiogram that was relieved by sublingual administration of nitroglycerin, no angiographic evidence of obstructive CAD after intracoronary nitroglycerin administration, and a positive result on intracoronary methylergonovine provocation testing. The control group consisted of patients who presented with atypical chest pain, no angiographic evidence of obstructive CAD, and negative results on intracoronary methylergonovine provocation testing (no CAS). Atypical chest pain was defined as spontaneous chest pain at rest and/or provoked by exertion that was relieved by sublingual administration of nitroglycerin [20] but not associated with ST-segment change on resting electrocardiogram. Exclusion criteria included the presence of obstructive CAD, coronary microvascular spasm [21], inflammatory manifestations probably associated with noncardiac diseases (e.g., infections and autoimmune disorders), liver disease/renal failure (serum creatinine level >2.5 mg/dL), collagen disease, malignancy, and loss of blood samples.

### Collection of Data

At baseline, we collected data on demographic information, coexisting illnesses, anthropometric values, use of medications, and laboratory values. Current smoking was defined as having smoked a cigarette within 3 weeks of the cardiac catheterization. Diabetes mellitus was diagnosed by fasting glucose ≥126 mg/dL on >2 occasions or defined from dietary treatment and/or medical therapy. Baseline seated blood pressure was the mean of 6 readings obtained during the first 2 office visits performed 2 weeks apart. Hypertension was defined as blood pressure of >140/90 mmHg on >2 occasions or receiving antihypertensive treatment.

### Laboratory Analysis

Blood specimens were collected after an overnight fast immediately before coronary angiography. Serum hs-CRP was measured in duplicate by an enzyme-linked immunosorbent assay (IMMULITE hs-CRP, Diagnostic Products Corp., Los Angeles, California). The lower limit of this assay was 0.10 mg/L and coefficients of variation were ≤5% at 0.20 mg/L of C-reactive protein.

### Provocative Protocol

Coronary angiography was performed using the standard Judkins technique. Nitrates and calcium antagonists were withdrawn for ≥24 hours before the procedure. Left ventricular ejection fraction was calculated using Simpson’s method. Obstructive CAD was defined as a ≥50% reduction in luminal diameter after administration of intracoronary nitroglycerin [22]. If no obstructive CAD was found, intracoronary methylergonovine (Methergin®; Novartis, Basel, Switzerland) was administered stepwise (1, 5, 10, 30 µg) first into the right coronary artery and subsequently into the left coronary artery. CAS was defined as a >70% reduction in luminal diameter compared with postintracoronary nitroglycerin, with associated angina and/or ST depression or elevation [23]. Provocation testing was stopped with an intracoronary injection of 50–200 µg of nitroglycerin (Millisrol®; G. Pohl-Boskamp, Hohenlockstedt, Germany).

### Follow-up

Patient follow-up data were obtained from medical records of outpatient visits and hospital readmissions or telephone interviews for patients with missing data. The study end point was the occurrence of a major adverse cardiovascular event, a composite of death, nonfatal myocardial infarction, and recurrent angina pectoris requiring repeat coronary angiography. All deaths were considered cardiac-related unless an unequivocal noncardiac cause could be identified. Coronary events were defined as nonfatal myocardial infarction and recurrent angina pectoris requiring repeat coronary angiography.

### Statistical Analysis

Continuous variables are expressed as mean± standard deviation or median value and 25th–75th percentiles, and log transformation was performed for variables with positive skewness for the subsequent Student’s t tests between groups. Categorical variables were analyzed using the χ^2^ test. Tertiles of hs-CRP were categorized as lowest (<1 mg/L), middle (1–3 mg/L), or highest (>3 mg/L) [16]. Multivariate-adjusted odds ratios (OR) and 95% confidence intervals (CI) calculated with multiple logistic regression were used to identify risk factors for CAS in patients without obstructive CAD. Model selection was based on a priori knowledge and the significance of univariate tests of the variables. Stratified analyses were performed on a subset of 555 patients with hs-CRP measurements to examine the interactions of hs-CRP tertiles and diabetes mellitus (model 1), and hypertension (model 2) on CAS. Differences in incidence of major adverse cardiovascular events and coronary event-free survival between groups during the follow-up period were assessed by the Kaplan-Meier method with the log-rank test. After univariate analysis, multivariate Cox regression analysis was carried out for the identification of independent predictors of outcome. We determined that 897 patients would provide a power of 90% to detect a 4.487-fold difference in the risk of developing a major adverse cardiovascular event between the two groups at an alpha level (two-sided) of 0.05. A two-sided p<0.05 was defined as statistically significant. All statistical analyses were performed with the statistical software package SPSS for Windows (Version 15.0, SPSS Inc., Chicago, IL).

## Results

**Figure 1:**
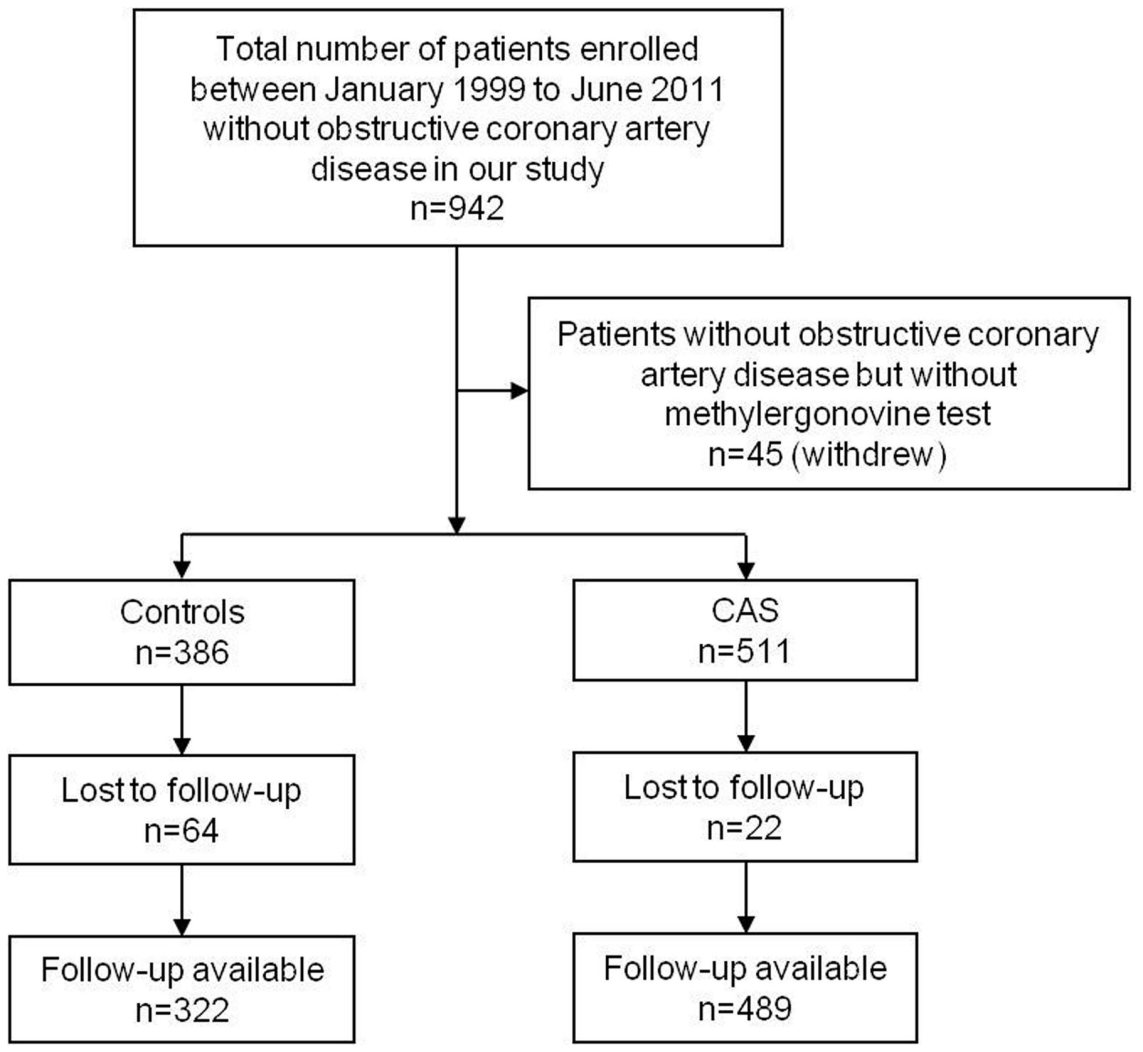
Flowchart of study subjects. CAS, coronary artery spasm.

Of all 897 patients from the initial study, follow-up data were available in 811 patients (90.4%), of whom 322 belonged to the control group (83.4% follow-up) and 489 belonged to the CAS group (95.7% follow-up) (Figure 1).

### Baseline Characteristics of Study Population

The mean age of the 897 patients was 57.1±11.7 years (range 19–88 years), and 39% were women. A total of 511 patients had CAS (spasm group) and 386 did not have CAS (control group). Advanced age, male gender, and current smoking status were associated with a greater likelihood of developing CAS. Moreover, hemoglobin level, hematocrit, platelet count, and hs-CRP were significantly higher in the CAS group than in the control group. Single vessel spasm was the most common finding in patients with CAS, and spasm was provoked mostly in the right coronary artery. The number of patients who used β-blockers and calcium channel blockers before coronary angiography was significantly greater in the control group than in the CAS group. However, after coronary angiography, the number of patients who used calcium channel blockers and nitrates was significantly greater in the CAS group than in the control group. There were no changes in diabetes treatments before and after coronary angiography (Table 1).

**Table 1:** Baseline characteristics.

Characteristics	Controls (n = 386)		CAS (n = 511)		p	
Age, years	56.1±11.4		57.8±11.9		0.030	
Men, n (%)	186 (48)		361 (71)		<0.001	
Body mass index, kg/m^2^	26.0±4.1		25.9±3.8		0.62	
Current smoker, n (%)	84 (22)		228 (45)		<0.001	
Diabetes mellitus, n (%)	70 (18)		98 (19)		0.69	
Hypertension, n (%)	178 (46)		221 (43)		0.39	
Left ventricular ejection fraction, %	67±12		66±9		0.10	
Hemoglobin A1c_,_ %	5.8±1.2		6.0±0.9		0.28	
Total cholesterol, mg/dL	202±41		198±41		0.14	
LDL cholesterol, mg/dL	137±39		133±44		0.29	
HDL cholesterol, mg/dL	38±14		37±14		0.80	
Hemoglobin, g/dL	13.2±2.0		13.9±1.6		<0.001	
Hematocrit, %	39.2±5.5		40.8±4.2		<0.001	
Platelet count, ×10^9^/L	209±71		220±65		0.030	
hs-CRP, median (25th–75th percentiles), mg/L*	1.19 (0.59–3.09)		2.02 (0.74–6.36)		<0.001	
Provoked coronary artery, n (%)						
Left main artery			4 (0.8)			
Left anterior descending artery			149 (29)			
Left circumflex artery			115 (23)			
Right coronary artery			341 (67)			
Number of provoked spastic artery, n (%)						
1-vessel spasm			418 (82)			
2-vessel spasm			35 (7)			
3-vessel spasm			58 (11)			
Medications, n (%)	A	D	A	D	A	D
β–blockers	69 (18)	35 (9)	51 (10)	26 (5)	0.001	0.020
Calcium channel blockers	174 (45)	151 (39)	148 (29)	485 (95)	<0.001	<0.001
Angiotensin Converting Enzyme inhibitors	39 (10)	66 (17)	51 (10)	92 (18)	0.95	0.73
Angiotensin receptor blocker	58 (15)	143 (37)	77 (15)	199 (39)	0.99	0.56
Nitrates	143 (37)	116 (30)	210 (41)	271 (53)	0.22	<0.001
Statins	46 (12)	77 (20)	66 (13)	118 (23)	0.66	0.26
Aspirin	266 (69)	124 (32)	337 (66)	143 (28)	0.35	0.18
Diuretics	93 (24)	42 (11)	102 (20)	77 (15)	0.14	0.07
Oral agents in diabetic patients	66 (94)		95 (97)		0.40	
Insulin in diabetic patients	1 (1)		2 (2)		0.77	
Diet only in diabetic patients	3 (4)		1 (1)		0.17	

Data are presented as mean± standard deviation unless mentioned otherwise. A, before angiography; CAS, coronary artery spasm; D, at discharge; hs-CRP, high-sensitivity C-reactive protein.

* hs-CRP samples were collected in a subset of 555 patients, with 223 and 332 in the control and CAS groups, respectively. Log-transformed values were used in analyses.

Among 350 women, current smoking status, hemoglobin, hematocrit, and hs-CRP levels were positively associated with CAS. Among 547 men, however, age, current smoking status, platelet count, and hs-CRP level were positively associated with CAS. Among patients with CAS, the prevalence of current smokers, hemoglobin and hematocrit levels were higher among men than women (Table 2).

**Table 2:** Gender-specific baseline characteristics between study groups.

Characteristic	Women (n = 350)			Men (n = 547)			
	Controls (n = 200)	CAS (n = 150)	p	Controls (n = 186)	CAS (n = 361)	p	p*
Age, years	58±10	58±11	0.90	54±12	58±12	0.002	0.88
Body mass index, kg/m^2^	25.8±4.2	25.8±3.7	0.94	26.2±3.9	25.9±3.8	0.39	0.72
Current smoker, n (%)	12 (6)	23 (15)	0.004	72 (39)	205 (57)	<0.001	<0.001
Diabetes mellitus, n (%)	43 (22)	31 (21)	0.85	27 (15)	67 (19)	0.24	0.58
Hypertension, n (%)	94 (47)	70 (47)	0.95	84 (45)	151 (42)	0.46	0.32
Left ventricular ejection fraction, %	68±12	67±10	0.64	67±12	65±9	0.19	0.05
Total cholesterol, mg/dL	205±42	202±45	0.50	199±40	197±39	0.45	0.18
Hemoglobin, g/dL	12.3±1.7	12.9±1.3	0.002	14.2±1.7	14.3±1.5	0.66	<0.001
Hematocrit, %	36.9±5.2	38.3±3.5	0.010	41.6±4.7	41.8±4.0	0.61	<0.001
Platelet, ×10^9^/L	213±82	221±75	0.38	206±58	220±61	0.020	0.86
hs-CRP, median (25th–75th percentiles), mg/L†	1.36 (0.71–3.08)	1.86 (0.88–6.92)	0.003	1.07 (0.48–3.21)	2.08 (0.71–6.03)	<0.001	0.45

Data are presented as mean± standard deviation unless mentioned otherwise. CAS, coronary artery spasm; hs-CRP: high-sensitivity C-reactive protein.

* Comparison between women and men among CAS patients.

† hs-CRP samples were collected in a subset of 555 patients, with 106 and 94 in the control and CAS groups in women, and 117 and 238 in the control and CAS groups in men, respectively. Log-transformed values were used in analyses.

### Gender-Specific Factors and CAS

Multivariate analysis of women revealed that while the highest hs-CRP tertile was independently associated with CAS, diabetes mellitus and hypertension were negatively associated with CAS (Table 3). However, age, current smoking status and the highest hs-CRP tertile were independently associated with CAS among men, with the highest hs-CRP tertile being the most significant factor.

**Table 3:** Gender-specific univariate and multivariate analysis of variables associated with CAS.

Variable	Units of increase	Women (n = 350)				Men (n = 547)				
		Univariate		Multivariate*		Univariate		Multivariate*		
		odds ratio(95% CI)	p	odds ratio(95% CI)	p	odds ratio(95% CI)	p	odds ratio(95% CI)	p	
Age	1 year	1.00 (0.98–1.02)	0.90	1.02 (0.99–1.05)	0.24	1.02 (1.01–1.04)	0.002	1.03 (1.00–1.05)	0.020	
Body mass index	1 kg/m^2^	1.00 (0.95–1.05)	0.94	1.00 (0.91–1.09)	0.95	0.98 (0.94–1.03)	0.39	1.02 (0.96–1.09)	0.50	
Current smoker	0 = no; 1 = yes	2.84 (1.36–5.91)	0.005	2.99 (0.93–9.62)	0.07	2.08 (1.45–2.99)	<0.001	1.83 (1.10–3.03)	0.020	
Diabetes mellitus	0 = no; 1 = yes	0.95 (0.57–1.60)	0.85	0.34 (0.13–0.88)	0.030	1.34 (0.83–2.18)	0.24	1.15 (0.59–2.26)	0.68	
Hypertension	0 = no; 1 = yes	0.99 (0.65–1.51)	0.95	0.46 (0.20–0.81)	0.010	0.87 (0.61–1.25)	0.46	0.96 (0.57–1.60)	0.87	
Left ventricular ejection fraction	1%	1.00 (0.98–1.01)	0.64	1.01 (0.98–1.03)	0.69	0.99 (0.97–1.01)	0.19	1.00 (0.98–1.02)	1.00	
Total Cholesterol	1 mg/dL	1.00 (0.99–1.00)	0.50	0.99 (0.99–1.00)	0.13	1.00 (0.99–1.00)	0.45	1.00 (0.99–1.00)	0.48	
Hemoglobin	1 g/dL	1.30 (1.10–1.53)	0.002	1.38 (0.93–2.06)	0.11	1.03 (0.91–1.17)	0.66	1.06 (0.60–1.87)	0.85	
Hematocrit	1%	1.07 (1.01–1.14)	0.020	0.97 (0.85–1.10)	0.61	1.01 (0.97–1.06)	0.61	0.99 (0.80–1.23)	0.94	
Platelet count	1×10^9^/L	1.00 (1.00–1.00)	0.38	1.00 (1.00–1.01)	0.47	1.00 (1.00–1.01)	0.020	1.00 (1.00–1.01)	0.32	
Tertile of hs-CRP										
<1 mg/L		1.0 (reference)		1.0 (reference)		1.0 (reference)		1.0 (reference)		
1–3 mg/L		0.97 (0.48–1.97)	0.93	0.76 (0.34–1.74)	0.52	1.27 (0.73–2.22)	0.39	1.13 (0.61–2.07)	0.71	
>3 mg/L		2.54 (1.28–5.01)	0.007	4.61 (1.99–10.67)	<0.001	2.48 (1.45–4.22)	0.001	2.02 (1.13–3.64)	0.020	

CAS, coronary artery spasm; hs-CRP: high-sensitivity C-reactive protein.

* Multivariate analysis was performed in a subset of 555 patients with hs-CRP measurements, with 200 in women, and 355 in men, respectively.

### Stratified Analyses of Hs-CRP Tertiles, and Diabetes Mellitus or Hypertension

Significant interactions were demonstrated between hs-CRP tertiles, and diabetes mellitus (model 1) or hypertension (model 2) for CAS risk in both genders (Table 4).

**Table 4:** Gender-specific stratified analysis for CAS of hs-CRP tertiles, and diabetes mellitus or hypertension.

Model			Women (n = 200)			Men (n = 355)		
			Tertile of hs-CRP			Tertile of hs-CRP		
			<1 mg/L	1–3 mg/L	>3 mg/L	<1 mg/L	1–3 mg/L	>3 mg/L
1	DiabetesMellitus	no	1 (reference)	0.67 (0.28–1.59)	4.41 (1.77–10.97)	1 (reference)	1.26 (0.66–2.41)	2.98 (1.53–5.84)
		yes	0.16 (0.01–1.88)	0.48 (0.07–3.35)	1.45 (0.48–4.34)	5.02 (1.03–24.54)	1.85 (0.45–7.60)	1.52 (0.64–3.63)
2	Hypertension	no	1 (reference)	1.43 (0.51–3.98)	9.68 (2.63–35.68)	1 (reference)	1.08 (0.49–2.38)	2.60 (1.15–5.92)
		yes	1.05 (0.36–3.09)	0.26 (0.06–1.02)	2.43 (0.88–6.75)	1.11 (0.51–2.41)	1.32 (0.53–3.29)	1.75 (0.81–3.81)

Data are presented as multivariate-adjusted odds ratio (95% confidence interval), with adjusted variables including age, body mass index, smoking, diabetes mellitus, hypertension, left ventricular ejection fraction, cholesterol, hemoglobin, hematocrit and platelet other than the stratified variable *per se.* CAS, coronary artery spasm; hs-CRP: high-sensitivity C-reactive protein.

1. **Model 1 analysis.** Non-diabetic women with the highest hs-CRP tertile had a 4.4-fold higher risk of developing CAS than those with the lowest hs-CRP tertile. Non-diabetic men with the highest hs-CRP tertile had a 3.0-fold higher risk of developing CAS than those with the lowest hs-CRP tertile. The ORs of CAS in women and men with the highest hs-CRP tertile reduced from 4.41 to 1.45 and 2.98 to 1.52, respectively, if they had diabetes mellitus. However, diabetes mellitus was a significant risk factor in men with the lowest hs-CRP tertile, among which diabetic men had a 5.0-fold higher risk for developing CAS than non-diabetic men. The prevalence of smoking in patients with CAS did not differ between those with and those without diabetes mellitus among women (18% vs. 10%; p = 0.40) or men (66% vs. 55%; p = 0.10).
2. **Model 2 analysis.** Non-hypertensive women with the highest hs-CRP tertile had a 9.7-fold higher risk for developing CAS than those with the lowest hs-CRP tertile. Non-hypertensive men with the highest hs-CRP tertile had a 2.6-fold higher risk for developing CAS than those with the lowest hs-CRP tertile. The ORs of CAS in women and men with the highest hs-CRP tertile reduced from 9.68 to 2.43 and 2.60 to 1.75, respectively, if they had hypertension. The prevalence of smoking in patients with CAS did not differ between those with and those without hypertension among women (16% vs. 15%; p = 0.90) or men (54% vs. 59%; p = 0.31).

### Stratified Analyses of Diabetes Mellitus and Hypertension

**Figure 2:**
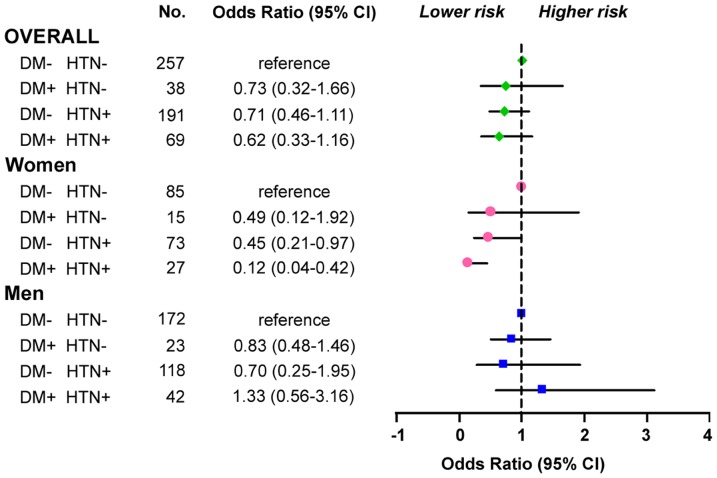
Multivariate-adjusted association of DM and HTN with risk of CAS according to different models. The odds ratios in the overall study population, women and men are represented by diamonds, circles and squares, respectively. The horizontal lines represent the 95% confidence intervals (CI). Adjusted logistic regression variables include age, body mass index, smoking, left ventricular ejection fraction, cholesterol, hemoglobin, hematocrit, platelet and hs-CRP tertiles other than the stratified variable *per se*. CAS, coronary artery spasm; DM, diabetes mellitus; hs-CRP, high-sensitivity C-reactive protein; HTN, hypertension.

Regardless of hs-CRP levels, both diabetes mellitus and hypertension appeared to be associated with a lower incidence of CAS in women and men (Figure 2). While women with diabetes mellitus and hypertension had the lowest risk of developing CAS among patients without obstructive CAD, hypertension had a more marked negative effect on the occurrence of CAS in diabetic patients (OR 0.12/0.49 = 0.24) than in their non-diabetic counterparts (OR 0.45/1 = 0.45). However, this effect was not observed in men.

### Predictive Factors

Univariate Cox regression analysis revealed that the highest hs-CRP tertile was a predictor of major adverse cardiovascular events and coronary events. After multivariate Cox regression analysis, the highest hs-CRP tertile remained a significant predictor. Diabetes mellitus and hypertension had no significant impact on major adverse cardiovascular events or coronary events (Table 5).

**Table 5:** Univariate and multivariate Cox regression analysis for major adverse cardiovascular events and coronary events.

	Univariate		Multivariate	
	Hazard Ratio (95% CI)	p	Hazard Ratio (95% CI)	p
Model 1: major adverse cardiovascular events				
Age (per 1 year)	0.987 (0.966–1.009)	0.26	0.986 (0.955–1.019)	0.40
Male sex (yes vs. no)	1.700 (0.933–3.097)	0.08	1.553 (0.546–4.412)	0.41
Current smoker (yes vs. no)	1.472 (0.852–2.545)	0.17	1.202 (0.477–3.031)	0.70
Diabetes mellitus (yes vs. no)	1.289 (0.676–2.457)	0.44	0.553 (0.187–1.638)	0.29
Hypertension (yes vs. no)	1.121 (0.650–1.934)	0.68	1.345 (0.596–3.033)	0.48
Left ventricular ejection fraction (per 1% )	0.991 (0.968–1.015)	0.46	1.006 (0.968–1.046)	0.76
Tertile of hs-CRP				
<1 mg/L	1 (reference)		1 (reference)	
1–3 mg/L	1.092 (0.220–5.421)	0.91	1.166 (0.232–5.866)	0.85
>3 mg/L	4.448 (1.311–15.092)	0.020	4.535 (1.287–15.980)	0.019
Model 2: coronary events				
Age (per 1 year)	0.983 (0.961–1.006)	0.16	0.981 (0.949–1.013)	0.24
Male sex (yes vs. no)	1.632 (0.874–3.051)	0.13	1.838 (0.604–5.597)	0.28
Current smoker (yes vs. no)	1.500 (0.844–2.666)	0.17	1.219 (0.475–3.128)	0.68
Diabetes mellitus (yes vs. no)	0.881 (0.411–1.884)	0.74	0.420 (0.123–1.427)	0.16
Hypertension (yes vs. no)	1.157 (0.652–2.052)	0.62	1.305 (0.571–2.985)	0.53
Left ventricular ejection fraction (per 1% )	0.993 (0.968–1.018)	0.57	1.006 (0.966–1.047)	0.78
Tertile of hs-CRP				
<1 mg/L	1 (reference)		1 (reference)	
1–3 mg/L	1.080 (0.218–5.361)	0.93	1.193 (0.237–6.017)	0.83
>3 mg/L	4.147 (1.216–14.137)	0.020	4.415 (1.241–15.712)	0.022

CI, confidence interval; hs-CRP: high-sensitivity C-reactive protein.

### Follow-up Data

**Figure 3:**
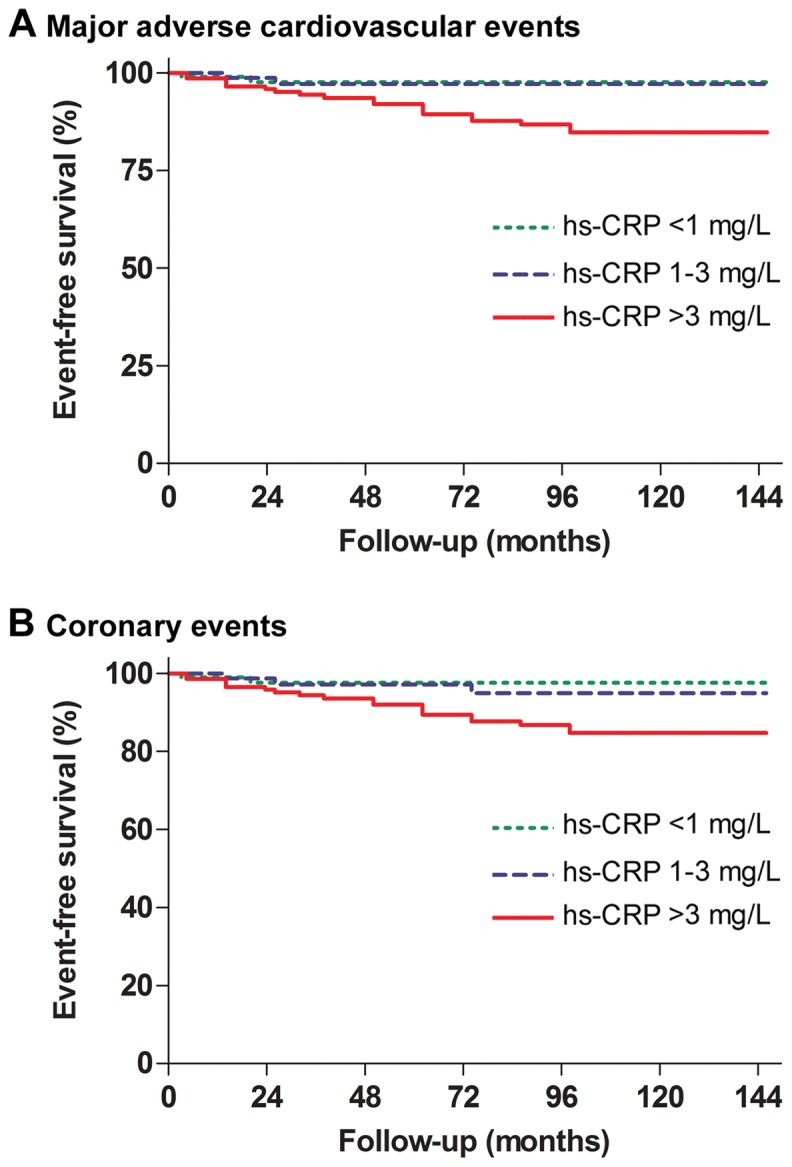
Outcome in patients with CAS in relation to hs-CRP tertiles. (A) Kaplan-Meier survival curves for major adverse cardiovascular event-free survival showing the frequency was lowest in patients with the highest hs-CRP tertile (p = 0.010) (log-rank test). (B) Coronary events showing significantly more events in patients with the highest hs-CRP tertile (p* = *0.021) (log-rank test). CAS, coronary artery spasm; hs-CRP, high-sensitivity C-reactive protein.

All patients with the exception of those who died were followed up for at least 12 months (range 1–150 months; mean 94±42 months; median 102 months). Three deaths occurred in the CAS group (0.6%), and the deaths were due to sudden cardiac death in 2 patients and due to cancer in 1 patient. Coronary events occurred in 40 (8%) patients in the CAS group. Of those patients, 36 had hs-CRP levels in the highest tertile, 3 had hs-CRP levels in the middle tertile, and 1 had a hs-CRP level in the lowest tertile. Three patients with the highest hs-CRP tertile in the CAS group had nonfatal myocardial infarction events (0.6%). Patients with the highest hs-CRP tertile had the lowest frequency of major adverse cardiovascular event-free survival and shortest recurrence-free survival of coronary events among the 3 hs-CRP tertile groups (Figure 3A and B, respectively).

## Discussion

We found that, among patients without obstructive CAD, diabetic women and diabetic men with low hs-CRP levels had a low and high risk of CAS, respectively. There were negative effects of diabetes mellitus and hypertension on CAS development in patients with high hs-CRP levels. Regardless of hs-CRP levels, hypertension had a more marked negative effect on CAS development in diabetic than in non-diabetic women, which was not observed in men. Nonetheless, an elevated hs-CRP was a strong and independent predictor of death, nonfatal myocardial infarction, and recurrent angina pectoris.

C-reactive protein is occasionally elevated in patients with diabetes mellitus [24]. Our finding that men but not women with diabetes mellitus who have low hs-CRP levels are at high risk of developing CAS indicates that gender differences in vascular reactivity [25] also extend to the diabetic state. In diabetic humans, endothelial cell dysfunction is characterized not only by decreased nitric oxide levels but also by increased synthesis of vasoconstrictors [26]. Therefore, this gender interaction in the association between hs-CRP and diabetes mellitus in CAS development suggests that hormones may play a role, especially in patients with low hs-CRP levels. Similar to a previous report [27], our findings suggest that the predictive value of hs-CRP elevations may vary among subsets of populations. In our study, diabetes mellitus and high hs-CRP levels had a negative effect on the development of CAS. Thus, there appears to be an interaction between hyperglycemia and high hs-CRP levels beyond each factor alone in CAS development. However, a causal role for hs-CRP is less certain and hs-CRP may be an epiphenomenon in CAS development.

Consistent with previous studies [2], [7], [19], while hypertension is negatively associated with CAS, we further demonstrate that this effect is more prominent in diabetic women than in their non-diabetic counterparts, which is not observed in men. Hypertension is associated with decreased contractile responses of coronary arteries to serotonin in rat coronary arteries [28]. While it has been demonstrated that synthetic phenotype smooth muscle cells are the main cultured vascular smooth muscle cell type from spontaneously hypertensive rats [29], contractile rather than synthetic phenotype smooth muscle cells play a main role in the pathogenesis of CAS. As a result, the negative association between CAS and hypertension indicates that two different vascular pathologies exist in CAS and hypertension, especially in women. Although diabetes mellitus has impaired vasoconstriction in response to different vasoconstrictors [30], [31], data on the effects of diabetes mellitus on the vascular response to acetylcholine, an inducer of CAS, however, are discrepant, which may be due to the differential effects of diabetes mellitus on the vascular bed studied [32].

Few studies in humans are available regarding the underlying mechanisms for the gender differences in the risk impact of hypertension on coronary dynamic abnormalities in patients with or without diabetes mellitus. While Lynch et al [33] showed a significant reduction in the distensibility of small coronary arteries from atrial appendage among hypertensive nondiabetic patients but not hypertensive diabetic patients, Schofield et al [34] showed that the distensibility of small subcutaneous arteries is increased in hypertension and is further increased in hypertension with diabetes mellitus. Although the influence of gender has not been assessed in the previous studies, it is, however, reasonable to speculate from animal studies that sex hormones are directly or indirectly involved. Sequencing of rat L-type calcium channel gene promoter region has provided evidence for a hormone response element strongly activated by testosterone [35]. Conversely, estrogen inhibits rat L-type calcium channel [36]. Furthermore, a previous study on hypertensive diabetic rats has shown that male rats exhibit an attenuated systemic vasodilator response to acetylcholine compared with the female rats [37]. Together, these data suggest that compared with males, females may be associated with less coronary spastic activity in the presence of hypertension and diabetes mellitus compared with hypertension alone; further studies are needed to address this issue.

To better understand the roles that diabetes mellitus, hypertension and hs-CRP play in CAS development, we included 894 patients with obstructive CAD during the same time period and performed a multiple logistic regression analysis to demonstrate the association of diabetes mellitus, hypertension and hs-CRP with obstructive CAD and CAS. Our results showed that the associations of diabetes mellitus and hypertension with the occurrence of obstructive CAD were significantly stronger than with that of CAS (OR: 3.35, 95% CI: 2.31–4.86 and OR: 1.71, 95% CI: 1.20–2.44, respectively), suggesting that the risks of obstructive CAD conferred by diabetes mellitus and hypertension are significantly higher than that of CAS. Furthermore, compared to diabetic men, who have a 2-fold to 3-fold increased risk of obstructive CAD, diabetic women are reported to have a 3-fold to 7-fold increased risk [38]. The risk of obstructive CAD associated with hypertension is also reported to be greater in women than in men [39]. Therefore, contrary to the results of our study in CAS, female gender does not reduce the risk of developing CAD in terms of diabetes mellitus and hypertension.

On the other hand, there was no statistical difference in the association between high hs-CRP level and disease type (obstructive CAD vs. CAS) (OR: 1.19, 95% CI: 0.79–1.79). Although in our study baseline hs-CRP levels in CAS patients were similar between genders, the OR for developing CAS was higher among women than among men in the highest hs-CRP tertile (4.61 vs. 2.02). In patients with obstructive CAD, hs-CRP was found to be a predictor of obstructive CAD [40]. Furthermore, similar to our findings, the relative risk for cardiovascular events associated with C-reactive protein was found to be higher for women than for men [41]. Based on these findings, we suggest that inflammation contributes to the development of both obstructive CAD and CAS without obstructive CAD. However, the strength of association was different between genders, which deserves prospective investigations.

Among our patients in the control group, possible mechanisms of onset of myocardial ischemia include (1) steal phenomenon resulting from reduction in coronary microvessel diastolic function or uneven vasodilation in the left ventricular wall, and (2) coronary microvascular spasm [21]. Our study excluded patients with coronary microvascular spasm, Therefore, in patients with microvascular angina, impairment of metabolic vasodilation in the coronary microvessels can cause ischemia in some regions of the myocardium or subendocardium [21].

Although age and left ventricular ejection fraction were identified as predictors of adverse prognosis in patients with CAS [42], the prognostic utility of hs-CRP has not been evaluated. We demonstrated for the first time that the highest hs-CRP tertile predicted future major adverse cardiovascular events and coronary events in patients with CAS and that event-free survival was significantly lower in those with elevated hs-CRP levels. Therefore, CAS patients with hs-CRP levels >3 mg/L should be considered for risk factor modification and aggressive treatment.

There are some limitations in this study. First, our study is of an observational nature, which may be explained by confounding. Therefore, we tried to control for confounding factors using multivariate modeling. The possibility of residual or undetected confounding is small but cannot be ruled out completely. Second, this study is based on a hospital patient cohort, and not a population-based causality analysis. The observational study design, even with extensive multivariable analysis, cannot prove causal relationships. Third, the sample size of women who were current smokers was relatively small, which might have resulted in an underestimation of the effect of smoking on risk for CAS in women. Fourth, the data were collected from community based teaching hospitals. One of the major limitations might be placed as the Berkerson’s bias under a referral delivery system. In addition, the patients’ behavior may be different as opposed to those in the primary care settings. Finally, we did not measure estrogen or androgen levels in our study patients. Therefore, whether the gender differences observed in our study are secondary to hormonal effects remain to be elucidated.

### Conclusions

Among patients without obstructive CAD, diabetes mellitus contributes to CAS development in men with low hs-CRP levels, but not in women. There are negative effects of diabetes mellitus and hypertension on CAS development in patients with high hs-CRP levels and especially in women. Nevertheless, elevated hs-CRP level is a strong and independent predictor of adverse prognosis in patients with CAS. Taken together, these suggest that gender, hs-CRP, diabetes mellitus, and hypertension should be incorporated into future diagnostic strategies aimed at earlier detection and management of CAS.
